# Campus Close-Down Came in Handy: Depression, Anxiety and Stress Among Sudanese Medical Students and Their Association With Brief and Extended Closure Periods

**DOI:** 10.1192/bjo.2022.212

**Published:** 2022-06-20

**Authors:** Hamid Magzoub, Mohammed Osman, Mohamed Mohamed

**Affiliations:** University of Khartoum Faculty of Medicine, Khartoum, Sudan

## Abstract

**Aims:**

To estimate the prevalence of depression, anxiety and stress among Sudanese medical students and investigate the relationship and impact of closure periods on depression.

**Methods:**

A cross-sectional analytical study was conducted. Data were collected from 1676 enrolled students from 10 medical schools in the capital of Sudan - Khartoum- who faced complete lockdown and agreed to participate in online google form. A self-administered questionnaire containing depression anxiety and stress scale (DASS-42) and socio-demographic inquires was used.

**Results:**

The chief responders were females 1158 (69.1%) while males were 518 (30.9%). The prevalence of stress symptoms was the highest (51.9%), followed by depression symptoms (49.8%) and anxiety symptoms (28.8%). 96 students attempted suicide (6%) and about 5 folds have suicidal ideation (27%). According to multiple binary logistic analyses, college closure time was significantly associated with decreasing chances of getting depression symptoms (OR: 0.39, 95% C.I: 0.21–0.70, p = 0.002), while being a female, COVID-19 patient or having a family history of depression appeared to elevate depression, anxiety and stress levels. The impact of university closure on stress and anxiety is non-significant.

**Conclusion:**

Depression, stress, and to a lesser extent anxiety are widespread among Sudanese medical students and suicidal ideation is noteworthy. All of these require serious and expeditious interventions. Controversially, our findings suggest that university closure serves as a protective factor, relieving depression in medical students but not exaggerating it.

